# Impact of Sodium‐Glucose Co‐Transporter 2 Inhibitors on Atrial Fibrillation Recurrence Post‐Catheter Ablation Among Patients With Type 2 Diabetes Mellitus: A Systematic Review and Meta‐Analysis

**DOI:** 10.1111/jce.16544

**Published:** 2025-01-09

**Authors:** Naser A. Abdelhadi, Khaled Mohamed Ragab, Mohammed Elkholy, Jayanthi Koneru, Kenneth A. Ellenbogen, Ajay Pillai

**Affiliations:** ^1^ Division of Cardiac Electrophysiology Virginia Commonwealth University Richmond Virginia USA; ^2^ Faculty of Medicine Minia University Minia Egypt; ^3^ Department of Radiology Beth Israel Deaconess Medical Center/Harvard University Boston Massachusetts USA

**Keywords:** atrial fibrillation, catheter ablation, sodium‐glucose co‐transporter 2 inhibitors, type 2 diabetes mellitus

## Abstract

Atrial fibrillation (AF) is the most common cause of arrhythmia‐induced cardiomyopathy. Effective management strategies include medical therapy for rate and rhythm control, catheter ablation (CA), and goal‐directed medical therapy. Sodium‐glucose co‐transporter 2 inhibitors (SGLT2i), a novel class of antidiabetic drugs, have shown a promising impact in reducing cardiovascular events in diabetic and nondiabetic heart failure (HF) patients. It is unclear what impact SGLT2i use may have on AF recurrence following CA. To evaluate the effects of SGLT2i on preventing AF recurrence following CA and its impact on other cardiovascular outcomes. We performed a comprehensive literature search through multiple search engines (PubMed, Scopus, Web of Science, and Cochrane) to include eligible studies using the appropriate keywords until 10 April 2024. Our search yielded nine eligible studies with 16 857 patients. Our analysis reveals a significant reduction in AF recurrence after CA among patients receiving SGLT2i compared to non‐SGLT2i medications (RR = 0.72, 95% CI [0.67–0.78], *p* < 0.00001). Additionally, SGLT2i therapy was associated with decreased all‐cause hospitalizations and reduced risk of ischemic stroke. However, no significant difference in all‐cause mortality was observed between SGLT2i and non‐SGLT2i groups. Our study found that SGLT2 inhibitors significantly reduced AF recurrence post‐CA in diabetic patients. Moreover, SGLT2i use was associated with lowered hospitalization and ischemic stroke risk. Though no significant difference in mortality was noted, the decrease in hospitalization suggests a possible favorable effect on cardiovascular events.

## 1 Introduction

Atrial fibrillation (AF) is the most common cause of arrhythmia‐induced cardiomyopathy [1]. AF, through rapid and irregular ventricular rates, may often be responsible for the development of cardiomyopathy [2], contribute to worsening of the cardiomyopathy in the setting of concomitant structural or ischemic heart disease [3], or represent sequelae of chronically elevated left atrial pressure and atrial remodeling due to cardiomyopathy [3].

Effective management of AF includes risk factor management, rate control, rhythm control, and stroke prevention [4]. The management of comorbidities, including diabetes mellitus, has been associated with favorable outcomes in regard to AF recurrence and cardiovascular burden such as heart failure (HF) and stroke risk [5]. Special considerations are present in patients with HF and reduced ejection fraction (HFrEF) [6]. Randomized clinical trials have demonstrated the benefit of catheter ablation (CA) of AF with pulmonary vein isolation (PVI) compared with medical therapy in patients with HFrEF [7].

Sodium‐glucose co‐transporter 2 inhibitors (SGLT2i) represent a relatively new class of oral antidiabetic drugs that act on the kidney to exert their effect through inhibition of glucose reabsorption [8]. A growing body of evidence supports their integral role in improving morbidity and mortality in HFrEF in both diabetic and nondiabetic patients [9]. While it has been suggested that SGLT2i reduces arrhythmia occurrence in HFrEF patients [10], it is unclear to what degree SGLT2i impacts AF recurrence following PVI [11].

This study aims to assess the association between SGLT2i use and AF recurrence following CA, as well as the potential impact of SGLT2i use on other cardiovascular events compared with non‐SGLT2i medications.

## 2 Methods

We conducted our study depending on the PRISMA [12] statement and the guidelines of the *Cochrane Handbook for Systematic Reviews* [13].

### 2.1 Searching Databases and Keywords

Four databases (PubMed, Scopus, Web of Science, and Cochrane) were searched until 10th April 2024 using the following key terms: (SGLT2 inhibitors OR sodium‐glucose cotransporter‐2 inhibitors OR SGLT2i OR canagliflozin OR dapagliflozin OR tofogliflozin OR empagliflozin OR Ertugliflozin OR Bexagliflozin) AND (atrial fibrillation OR AFib) AND ablation.

(“SGLT2 inhibitors” OR “sodium‐glucose cotransporter‐2 inhibitors” OR “SGLT2i” OR canagliflozin OR dapagliflozin OR tofogliflozin OR empagliflozin OR Ertugliflozin OR Bexagliflozin) AND (“atrial fibrillation” OR AFib) AND ablation (“SGLT2 inhibitors” OR “SGLT2i” OR canagliflozin OR dapagliflozin OR tofogliflozin OR empagliflozin OR Ertugliflozin OR Bexagliflozin) AND (“atrial fibrillation” OR AFib) AND ablation. Manual research was done in the reference records of the included studies to complete the electronic without any restrictions regarding the publication date or the language of the studies throughout the whole process.

### 2.2 Eligibility Criteria and Study Selection

All patients with type 2 DM who have undergone CA for AF (P) who received SGLT2i (I) compared with patients who received non‐SGLT2i medications (C) regarding the AF recurrence and other clinical outcomes (O) including any type of study design (S). Three authors screened titles and abstracts of the potentially included studies and then reviewed the full text to confirm their eligibility criteria. The supervisor was involved in clearing any case of indecision.

### 2.3 Data Extraction and Risk of Bias Assessment

Baseline items were extracted from the included studies by three independent authors including (a) study arms, sample size; (b) Demographics: sex, age, body mass index (BMI) (kg/m^2^) of patients; (c) AF characteristics: type, and duration; (d) Clinical parameters: HbA1c, CHA2DS2‐VASc score which is a clinical prediction tool utilized to estimate stroke risk in patients with AF, left atrial dimension (LAD); (e) Comorbidities: hypertension (HTN), cardiovascular diseases such as HF, ischemic heart disease (IHD), renal disease, lipid disorders, and thyroid disease; and (f) Medications: antiarrhythmic drugs, antidiabetic drugs (metformin and insulin). Another two authors extracted the following summary data including site, study design, total sample size, inclusion criteria, and conclusion.

The quality of the RCT was categorized into high, low, or unclear risk using the Cochrane tool for evaluating bias, as outlined in the *Cochrane Handbook for Systematic Reviews* [13]. NIH Quality Assessment Tool for Observational Cohort and Cross‐Sectional Studies was used to assess the risk of bias in included cohort studies.

#### 2.3.1 Definition of AF Recurrence

AF recurrence was defined mainly as an episode of atrial tachyarrhythmia lasting for > 30 s without antiarrhythmic drugs after a 3‐month blanking period. Recurrence as multiple events was also acknowledged.

### 2.4 Statistical Analysis

We conducted our meta‐analysis using Review Manager Software 5.4 (The Cochrane Collaboration, London, England). Continuous outcome data were pooled as mean differences (MD) accompanied by 95% confidence intervals (CIs), when different assessment tools were employed, we aggregated the data as standardized mean differences (SMD). Dichotomous outcomes were aggregated as risk ratios (RR) or hazard ratios (HR) with corresponding 95% CIs. Initially, a fixed‐effect model was employed to aggregate data, and heterogeneity between studies was evaluated using the Chi‐square (*χ* ^2^) and *I* ^2^ statistic. Upon detecting significant heterogeneity (*p* < .1 and *I* ^2^ > 50%), we adopted a random‐effect model. Sensitivity analysis, employing the leave‐one‐out method, was performed to solve heterogeneity. The Mantel–Haenszel (MH) test was applied to determine the effect sizes and to assess heterogeneity among the studies. Statistical significance was defined as a *p* value < 0.05.

## 3 Results

### 3.1 Literature Search

**Figure 1:**
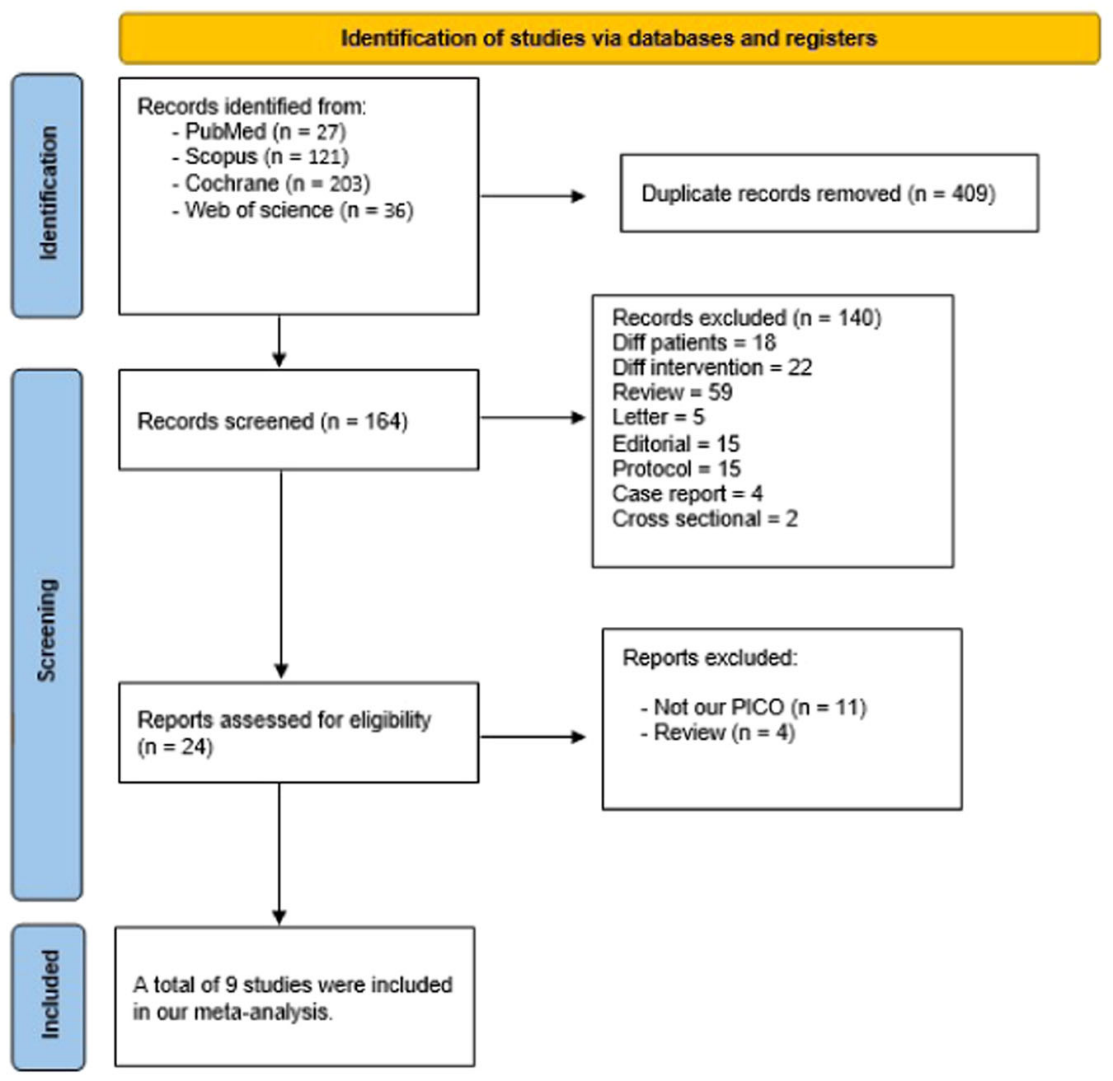
PRISMA flow diagram.

We conducted a comprehensive literature search across various search engines including (PubMed, Scopus, Web of Science, and Cochrane), resulting in 204 records. After eliminating 40 duplicate papers, 140 articles were excluded based on title and abstract screening, excluding any study not fitting our inclusion criteria such as case reports, case series, letters, reviews, and conference letters. Following the full‐text screening of 24 remaining articles, nine studies [11, 14, 15, 16, 17, 18, 19, 20, 21] met our criteria and proceeded to all stages of meta‐analysis to contribute evidence (See Figure 1).

### 3.2 Baseline Characteristics

Of 16 857 patients with type 2 diabetes who have undergone CA for AF enrolled in our study, 3820 patients were administered SGLT2i after initial CA, either radiofrequency ablation or cryoballoon ablation. A summary of the included studies is shown in Table 1. The baseline characteristics of the study cohorts are shown in Table 2, including general characteristics, demographics, AF characteristics, laboratory values, comorbidities, and medications.

**Table 1:** Summary of included studies.

ID	Site	Study design	Total sample size	Inclusion criteria	Conclusion	Follow‐up method
Abu‐Qaoud et al. [16]	USA	Retrospective cohort study	13 300	Patients ≥ 18 years with a history of type 2 DM.	“SGLT2—Is in patients with type 2 DM undergoing AF ablation is associated with a lower risk of needing subsequent cardioversion, new AAD therapy, and re‐do AF ablation. This suggests that SGLT2‐Is may increase the likelihood of maintaining sinus rhythm after AF ablation in patients with type 2 DM and AF”	Clinical, ECG, 24‐h Holter monitoring, cardiac device
Fichadiya et al. [19]	Canada	Retrospective cohort study	2242	Adults ≥ 18 years with both DM and AF.	“Among patients with concomitant DM and AF, the prescription of SGLT2i was associated with fewer AF events, lower risk of all‐cause mortality, and fewer HF‐related hospitalizations compared with DPP4i. While these results are consistent with the emerging data on the effects of SGLT2i on AF, future well‐powered clinical trials are required to confirm these associations, given a possible residual confounding”	
Kishima et al. [15]	Japan	Prospective, randomized controlled study	70	1. Type 2 DM (6.5% < glycated hemoglobin [HbA1c] < 10%) combined with AF 2. Treated with oral anticoagulants 3. Duration of AF < 1 year 4. Not taking SGLT2i and/or DPP4i for > 2 weeks before enrollment 5. Age 20–80 years	“Tofogliflozin and SGLT2i were associated with a significantly lower risk of recurrent AF”	ECG, 24‐h Holter monitoring, clinical
Liu et al. [18]	Taiwan	Retrospective cohort study	122	Patients with type 2 DM undergoing CA for antiarrhythmics–refractory AF.	“SGLT2i and AF type (paroxysmal) were independent risk factors associated with the absence of atrial tachyarrhythmia recurrence after CA in patients with type 2 DM and AF. The pleiotropic effects of SGLT2i on BMI reduction and left ventricular function improvement may play a role in preventing AF recurrence post‐CA”	ECG, 24‐h Holter monitoring, cardiac device
Luo et al. [17]	China	Retrospective cohort study	326	Patients with type 2 DM and drug‐refractory AF.	“Dapagliflozin could decrease the ATa recurrence after ablation. Furthermore, dapagliflozin was a significant predictor of the absence of ATa after ablation in the follow‐up”	
Noh et al. [21]	Korea	Retrospective cohort study	272	Patients with type 2 DM and drug‐refractory AF.	“DAPA was associated with a reduction in total atrial arrhythmia recurrence after ablation and SGLT2 inhibitors could protect against AF recurrence”	
Zhao et al. [11]	China	Prospective cohort study coupled with a meta‐analysis	525	Patients with AF aged ≥ 18 years undergoing initial CA with a history of diabetes.	“Lower risk of AF recurrence with the use of SGLT2i among patients with diabetes after AF ablation. Moreover, patients with larger BMI, persistent AF, and longer AF duration seem to benefit more from SGLT2i therapy after AF ablation. Further RCTs are warranted to fully assess the impact of SGLT2i on AF recurrence after CA in patients with or without diabetes”	Clinical, ECG, 24‐h Holter monitoring

**Table 2:** Baseline characteristics of cohorts within included studies.

ID	Intervention	Number		Demographics			AF characteristics				Laboratory values			Comorbidities, *N* (%)							Medications N (%)		
				Age, mean (SD)	Male, *N* (%)	BMI (kg/m^2^), Mean (SD)	Type, *N* (%)		Duration, mean (SD)	HbA1c, mean (SD)	CHA2DS2‐VASc score, mean (SD)	LVEF, mean (SD)	LAD (mm), mean (SD)	HTN	CVS disease			Renal disease	Lipid disorder	Thyroid disease	Cardiac	Antidiabetic	
			SGLT2i treatment vs. comparator arm				Paroxysmal	Persistent							HF	IHD	others				Antiarrhythmic	Metformin	Insulin
Abu‐Qaoud et al. [16]	SGLT2i	2326		65.3 ± 9.1	1730 (74.37)	NA	NA	NA	NA	NA	NA	NA	NA	2202 (93)	1449 (61)	1607 (68)	NA	630 (27)	2029 (86)	722 (31)	2170 (92)	1721 (73)	1738 (74)
	Non‐SGLT2i	10 974	Dapagliflozin vs. non‐SGLT2i medications (no specific medication listed)	65.8 ± 9.4	7166 (65.29)	NA	NA	NA	NA	NA	NA	NA	NA	8866 (81)	4313 (39)	5502 (50)	NA	1829 (16.4)	7564 (85)	2825 (26)	8423 (77)	4336 (40)	4255 (39)
Fichadiya et al. [19]	SGLT2i	1121		64.8 ± 10.2	844 (75.28)	NA	NA	NA	6.6 (4.8)	NA	2.2 ± 1.2	NA	NA	1030 (91.9)	393 (35.1)	158 (14.1)	42 (3.7)	912 (81.4)	NA	149 (13.3)	182 (16.2)	949 (84.7)	301 (26.9)
	DPP4i	1121		68.0 ± 10.5	817 (72.88)	NA	NA	NA	6.9 (4.8)	NA	2.4 ± 1.3	NA	NA	1055 (94.1)	494 (44.1)	180 (16.1)	77 (6.9)	941 (83.9)	NA	176 (15.7)	224 (20.0)	937 (83.6)	262 (23.4)
Kishima et al. [15]	Tofogliflozin	38	Tofogliflozin vs. anagliptin (DPP4i)	70.3 ± 8.6	26 (68.0)	25.5 ± 4.6	NA	NA	35.9 ± 50.4	6.7 ± 0.6	2.6 ± 1.1	61.5 ± 11.3	45.6 ± 7.5	26 (68.0)	10 (26.0)	2 (5.0)	21 (56)	NA	26 (68.0)	NA	4 (11)	8 (21.0)	1 (3.0)
	Anagliptin	32		70.3 ± 7.7	22 (69.0)	25.3 ± 4.3	NA	NA	41.5 ± 44.2	6.8 ± 0.9	2.7 ± 1.3	58.0 ± 15.4	44.7 ± 5.0	19 (59.0)	9 (28.0)	2 (6.0)	13 (41)	NA	19 (59.0)	NA	6 (19)	5 (16.0)	0
Liu et al. [18]	SGLT2i	45	Empagliflozin, dapagliflozin, canagliflozin vs. non‐SGLT2i medications (no specific medication listed)	60.1 ± 10.6	35 (78)	28.6 ± 4.2	32 (71)	13 (29)	3.09 ± 3.10	6.8 ± 0.7	2.8 ± 1.2	NA	NA	36 (80)	NA	10 (22)	9 (20)	NA	26 (58)	7 (16)	39 (118)	36 (80)	1 (2)
	Non‐SGLT2i	77		63.2 ± 8.6	54 (70)	27.3 ± 3.9	54 (70)	23 (30)	3.67 ± 3.73	6.6 ± 0.8	2.9 ± 1.3	NA	NA	57 (74)	NA	9 (12)	13 (17)	NA	47 (61)	9 (12)	51 (134)	54 (70)	5 (7)
Luo et al. [17]	Dapaglifozin	79		63.4 ± 10.4	48 (60.75)	25.9 ± 3.7	NA	35 (44.3)	NA	7.7 ± 1.4	NA	60.4 ± 6.7	40.2 ± 6.4	53 (67.0)	NA	31 (39.2)	NA	NA	21 (26.5)	NA	17 (21.6)	42 (53.2)	7 (8.9)
	Control	247		63.8 ± 9.9	144 (58.29)	26.7 ± 3.5	NA	119 (48.2)	NA	7.3 ± 1.2	NA	60.0 ± 7.2	41.0 ± 6.5	157 (63.6)	NA	102 (41.3)	NA	NA	50 (20.2)	NA	58 (23.5)	139 (56.3)	39 (15.8)
Noh et al. [21]	Dapaglifozin	73		73.47 ± 4.79	61 (83.6)	25.16 ± 3.64	43 (58.9)	30 (41.1)	NA	NA	NA	57.23 ± 7.26	43.94 ± 5.45	50 (68.5)	NA	3 (4.1)	NA	NA	NA	NA	73 (100)	NA	NA
	Control	199		71.72 ± 5.61	175 (87.9)	23.73 ± 5.45	107 (53.8)	92 (46.2)	NA	NA	NA	59.68 ± 6.18	45.35 ± 5.30	141 (70.9)	NA	33 (16.6)	NA	NA	NA	NA	198 (100)	NA	NA
Zhao et al. [11]	SGLT2i	138	Dapagliflozin, tofogliflozin, empagliflozin vs. non‐SGLT2i medications (no specific medication listed)	63.9 ± 8.7	98 (71.0)	26.4 ± 3.2	NA	64 (46.4)	3.5 ± 3.8	6.3 ± 1.0	2.9 ± 1.1	62.5 ± 7.3	41.8 ± 5.8	103 (74.6)	29 (21.0)	52 (37.7)	NA	NA	NA	NA	NA	66 (47.8)	21 (15.2)
	Non‐SGLT2i	387		64.0 ± 9.5	276 (71.3)	26.3 ± 3.4	NA	203 (49.6)	3.6 ± 5.2	6.4 ± 0.8	2.7 ± 1.5	63.2 ± 6.7	41.4 ± 5.2	314 (76.2)	57 (14.0)	127 (32.8)	NA	NA	NA	NA	NA	162 (41.9)	48 (12.4)

### 3.3 Risk of Bias

**Figure 2:**
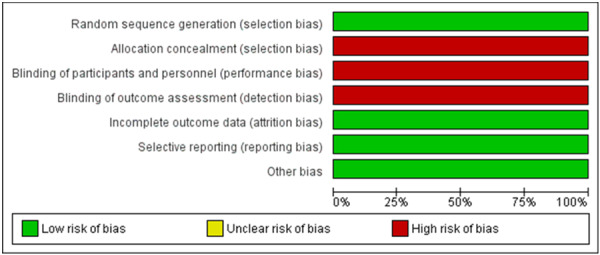
Risk of bias graph.

Assessment of the risk of bias was performed by two independent authors. The quality of the selected RCT was low biased in most domains but relatively high regarding selection, performance, and detection bias as shown in Figure 2. The cohort study's risk of bias assessment came fair in general except for three studies that were judged as poor.

### 3.4 Clinical Outcomes Analysis

#### 3.4.1 Primary Outcome

##### 3.4.1.1 AF Recurrence

**Figure 3:**
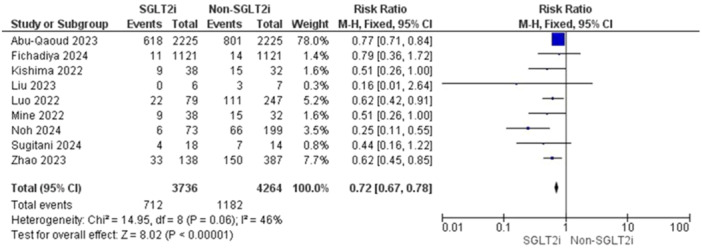
AF recurrence before solving heterogeneity.

**Figure 4:**
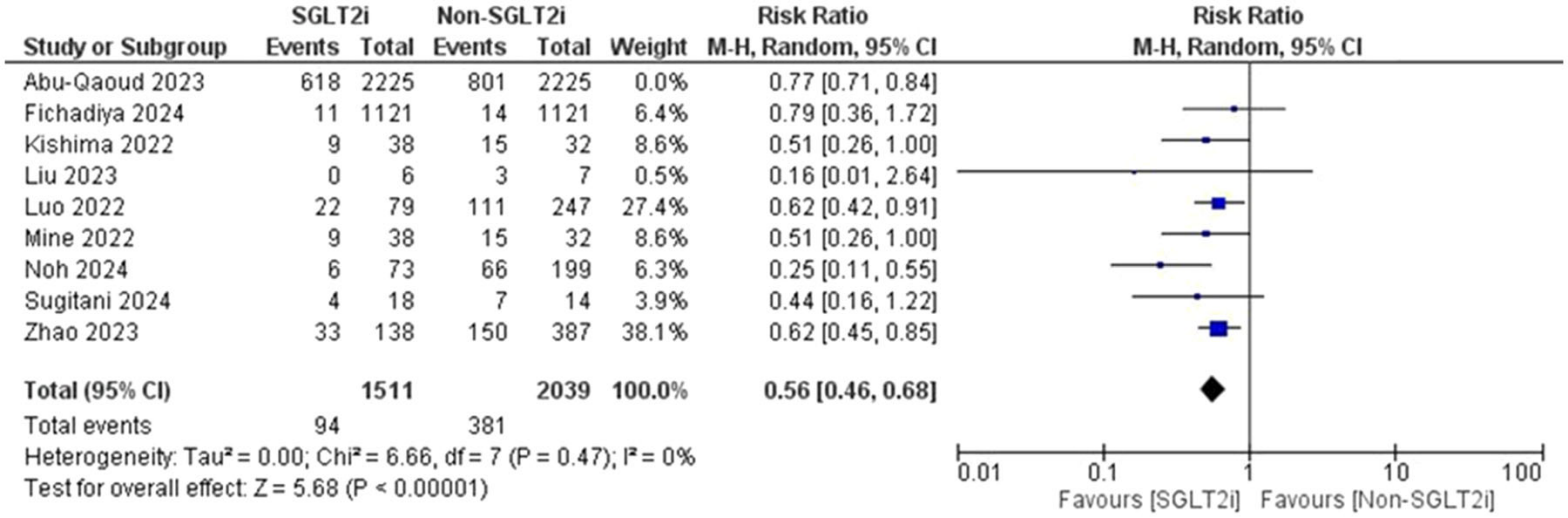
AF recurrence after solving heterogeneity.

Pooled data from nine studies resulted in 8000 participants, with 1894 total AF recurrences; 712 AF recurrences were found in the SGLT2i group with one included study that showed no AF recurrence and 1282 AF recurrences in the patients who did not receive an SGLT2i inhibitor (RR = 0.72, 95% CI [0.67–0.78], *p* < 0.00001) and the pooled analysis was heterogeneous (*p* = 0.06, *I* ^2^ = 46%) as shown in Figure 3. The heterogeneity could be resolved by the use of the leave one out method as the (*p* = 0.47, *I* ^2^ = 0%). The results were the same after excluding Abu‐Qaoud et al. showing the superiority of SGLT2i over the other drugs (RR = 0.56, 95% CI [0.46–0.68], *p* < 0.00001) as shown in Figure 4.

#### 3.4.2 Secondary Outcomes

##### 3.4.2.1 All Cause Hospitalization

**Figure 5:**
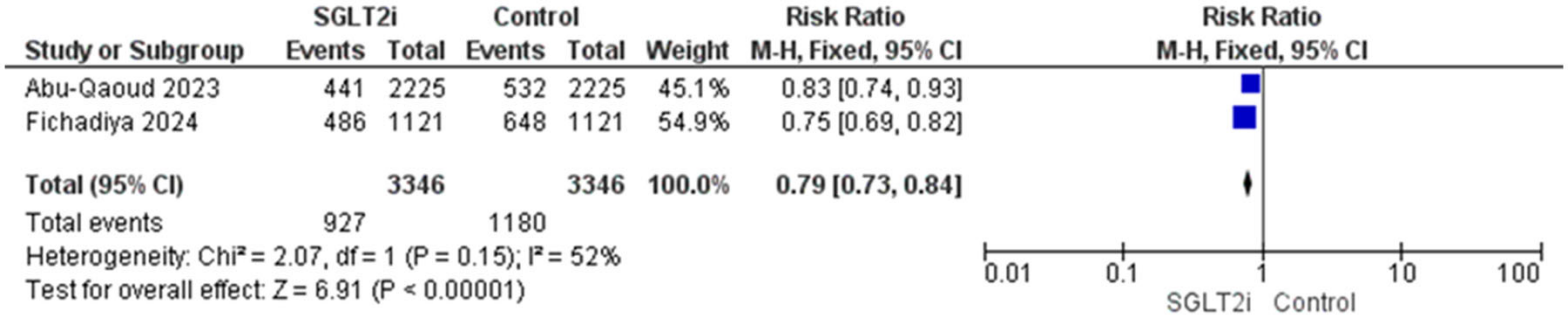
All‐cause hospitalization.

Two studies reported this outcome with 6692 patients [16, 19]. The overall estimate was significant showing a reduction in hospitalization in patients who received the SGLT2i (RR = 0.79, 95% CI [0.73–0.84], *p* < 0.00001), and the pooled analysis was homogenous (*p* = 0.15, *I* ^2^ = 52%) (Figure 5).

##### 3.4.2.2 All‐Cause Mortality

**Figure 6:**
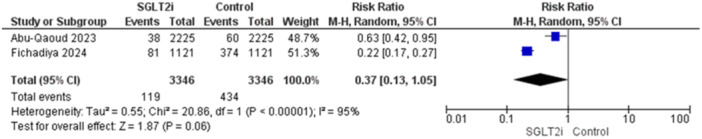
All‐cause mortality.

A total of 6692 patients from two trials reported this outcome [16, 19]. There was no significant difference between the SGLT2i group and the non‐SGLTi group (RR = 0.37, 95% CI [0.13–1.05], *p* = 0.06), and the pooled analysis was heterogenous (*p* < 0.0001, *I* ^2^ = 95%) (Figure 6).

##### 3.4.2.3 Ischemic Stroke

**Figure 7:**
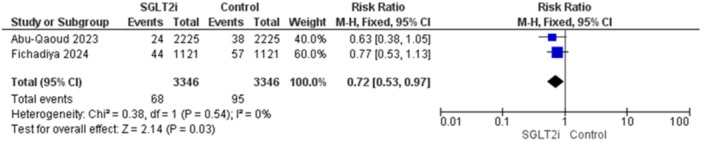
Ischemic stroke.

Ischemic stroke was reported in two trials [16, 19]. The overall analysis significantly favored patients who received SGLT2i inhibitors and the results were (RR = 0.72, 95% CI [0.53–0.97], *p* = 0.03), and the homogeneity between studies was observed (*p* = 0.54, *I* ^2^ = 0%) (Figure 7).

##### 3.4.2.4 AF‐Recurrence Risk Between Two Groups

**Figure 8:**
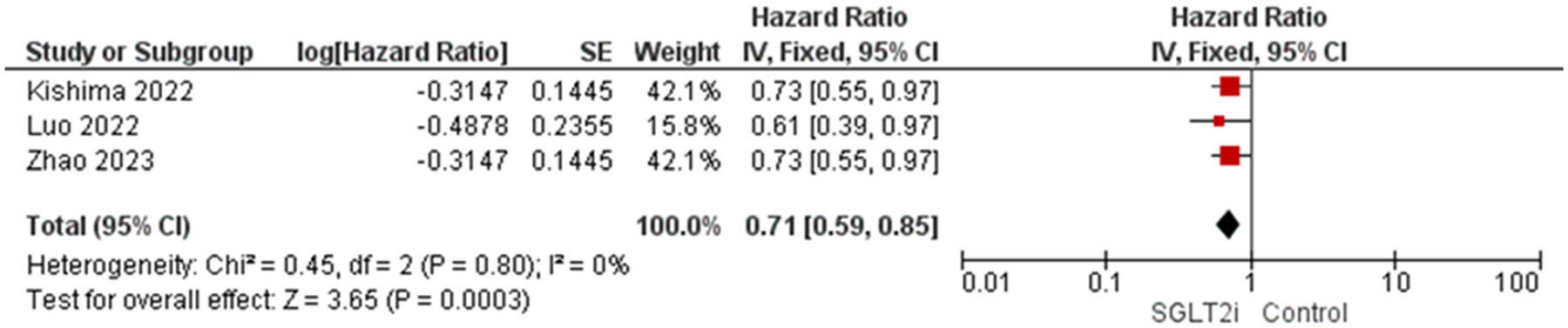
AF recurrence risk.

Three studies reported this outcome [11, 15, 17]. The overall analysis showed statistical significant toward SGLT2 inhibitors group as the results were (HR = 0.71, 95% CI [0.59–0.85], *p* = 0.0003), and the homogeneity between studies was observed (*p* = 0.80, *I* ^2^ = 0%) (Figure 8).

## 4 Discussion

The primary finding of our study showed a significant reduction in the primary outcome of AF recurrence risk among diabetic patients taking SGLT2 inhibitors (SGLT2i) compared to those not on non‐SGLT2i drugs, highlighting the potential benefit of SGLT2i. The hypothesis suggests that SGLT2 inhibitors reduce the likelihood of AF recurrence in patients following CA.

The precise mechanism by which SGLT2 inhibitors (SGLT2i) affect AF is incompletely understood. Electrophysiologic studies have demonstrated that SGLT2 inhibitors attenuate action potential duration prolongation in diabetic and HF models. Effects have been documented on various cardiac ion channels, including the late inward sodium current (*I*_Na‐late_), transient outward potassium channel (*I*_to_), slow delayed rectifier potassium current (*I*_Ks_), and l‐type calcium current (*I*_Ca‐L_), among others. SGLT2i use has additionally been associated with changes in mitochondrial homeostasis. SGLT2i use may decrease atrial fibrosis and hypertrophy, thus decreasing atrial structural changes which may contribute to AF maintenance [22]. The diuretic effect associated with SGLT2i use may lead to better control of blood pressure, intravascular blood volume and left atrial pressure [23].

Our findings suggesting a reduction in AF recurrence risk are similar to the results of other studies that concluded the potential benefit of SGLT2i in reducing AF recurrence after CA. While CA has been shown to result in superior outcomes compared to antiarrhythmic drugs in patients with paroxysmal AF, reported recurrence rates in the first year have ranged from 20% to 40% [18, 19, 20, 21]. A lower incidence of AF recurrence after CA was noted among patients with type 2 DM receiving SGLT2i compared with patients without SGLT2i in retrospective cohort studies [15, 16, 17]. Analyses of AF recurrence by subgroup (paroxysmal vs. persistent) was not possible in the current study.

A significant reduction in ischemic stroke risk in the SGLT2i group was observed. This finding may correlate with studies that reported reductions in blood pressure, body weight, and blood vessel wall stiffness associated with SGLT2i [24, 25]. SGLT2i treatment was associated with a notable decrease in all‐cause hospitalization. Moreover, a significant reduction in all‐cause hospitalization and ischemic stroke risk through decreasing cardiovascular events was observed.

AF recurrence was defined as an episode of atrial tachyarrhythmia lasting for > 30 s without antiarrhythmic drugs after a 3‐month blanking period. This definition was consistent across all studies. Most studies considered recurrence as a single event, but a few studies, such as Liu et al. [18] and Zhao et al. [11], considered repeated recurrence as multiple events and double‐counted them.

While a trend toward reduced mortality was observed in the SGLT2i group, there was no significant reduction in all‐cause mortality between the SGLT2i and non‐SGLT2i groups. This dissociation between treatment effects on HF hospitalization and cardiovascular mortality was also observed in prospective randomized clinical trials [16, 19].

Overall, our study sheds light on the potential benefits of SGLT2i on AF recurrence after CA in diabetic patients. Further investigation is warranted to confirm these findings and for a better understanding of the mechanisms of SGLT2i in reducing AF recurrence and improving cardiovascular outcomes.

While none of the studies directly assessed AF burden as a measure of procedural success, we can still glean valuable insights by analyzing the available data. Comparing AF duration and episode counts between the SGLT2i and control groups, along with assessing antiarrhythmic drug use and Holter monitoring results, might provide an indirect measure of procedural effectiveness. Subgroup analyses, where applicable, can further refine our understanding. However, the retrospective nature of the data, potential biases, and varying methodologies across studies necessitate caution. Despite these limitations, this analysis highlights the need for future prospective studies specifically designed to measure procedural success by assessing AF burden in relation to SGLT2i use.

The effect of SGLT2i among patients who have never undergone CA was discussed in several studies. One study reported that SGLT2i helped to reduce the risk of AF recurrence in patients with diabetes who had not undergone CA [19]. Another studies showed a lower incidence of AF in patients treated with SGLT2 inhibitors compared to those receiving a placebo [26, 27]. These results suggest that SGLT2i have potential benefits beyond glycemic control and are a promising therapeutic option for preventing AF recurrence.

Our study consisted of a high number of patients with a total of 16 857 from nine different studies. The heterogeneity of the included studies was relatively low, improving the quality of the analysis. The analysis is limited by the fact that the comparator groups are heterogenous across studies, with control groups consisting of either differing anti‐glycemic agents or no agent at all. This limits comparisons to real‐world practice. Additionally, Liu et al.'s [18] study reported a statistically significant benefit of SGLT2i use, the small sample size and potential for follow‐up bias warrant caution in interpreting the results, and larger studies with longer follow‐up are needed to confirm these findings. Subgroup analysis of different clinical subtypes of AF was not possible. The impact on AF recurrence in nondiabetic patients was not studied. Randomized control trials with larger patient populations are required to confirm these results.

## 5 Conclusion

SGLT2i use in diabetic patients was associated with significantly reduced AF recurrence risk following CA. Additionally, SGLT2i use was associated with decreased all‐cause hospitalization and ischemic stroke risk. Although no significant disparity in all‐cause mortality between SGLT2i and non‐SGLT2i groups was noted, the notable decrease in all‐cause hospitalizations is suggestive of benefit. Further randomized controlled trials are needed to better evaluate the effects of SGLT2i on AF recurrence following CA, regardless of diabetes status.

## Disclosure

The authors have nothing to report.

## Ethics Statement

The authors have nothing to report.

## Data Availability

The data that supports the findings of this study are available in the supplementary material of this article.
